# The development and evaluation of hyaluronic acid coated mitochondrial targeting liposomes for celastrol delivery

**DOI:** 10.1080/10717544.2022.2162156

**Published:** 2023-01-04

**Authors:** Simeng Xiao, Siying Huang, Xiaojing Yang, Yujie Lei, Mingxiang Chang, Junjie Hu, Yan Meng, Guohua Zheng, Xinyan Chen

**Affiliations:** a Pharmacy Faculty, Hubei University of Chinese Medicine, Wuhan, China; b Key Laboratory of Chinese Medicine Resource and Compound Prescription, Ministry of Education, Hubei University of Chinese Medicine, Wuhan, China; c Pharmacy Department, Wuxue No.1 People’s Hospital, Wuxue, China; d Laboratory of Cell and Molecular Biology, Hubei Hospital of Traditional Chinese Medicine, Wuhan, China

**Keywords:** Celastrol, mitochondrial targeting, tumor targeting, delivery system, anticancer effect

## Abstract

In order to precisely deliver celastrol into mitochondria of tumor cells, improve antitumor efficacy of celastrol and overcome its troublesome problems in clinical application, a novel multistage-targeted celastrol delivery system (C-TL/HA) was developed via electrostatic binding of hyaluronic acid (HA) to celastrol-loaded cationic liposomes composed of natural soybean phosphatidylcholine and cholesterol modified with mitochondrial targeting molecular TPP. Study results in this article showed that C-TL/HA successfully transported celastrol into mitochondria, effectively activated apoptosis of mitochondrial pathway, exerted higher tumor inhibition efficiency and lower toxic side effects compared with free celastrol. More importantly, HA coating not only enabled this delivery system to have good stability and safety *in vivo*, but also increased drug uptake and facilitated tumor targeting through recognizing CD44 receptors rich on the surface of tumor cells. Conclusively, this HA-coated mitochondrial targeting liposomes may provide a prospect for the clinical application of celastrol in tumor therapy.

## 1. Introduction

Celastrol (Cela) is a natural bioactive ingredient obtained from Tripterygium, a traditional Chinese medicine mainly used to treat rheumatoid arthritis. Recently, increasing attention has been focused on the anti-cancer property of Cela, and promising results have proved that Cela plays an anti-carcinogenic role in many malignant tumors (Li et al., 2020; Qin et al., 2020; Zhu et al., 2020) through multiple mechanisms (Li et al., 2015, 2019). Cela has been rated as the top-five prospective cancer-specific natural drug, which may become a potential candidate for clinical cancer therapy. However, it still suffers from many disadvantages, including poor water solubility, low bioavailability, and severe toxic side effects derived from undesirable systemic distribution, which are the main challenges to be addressed in clinical applications.

Apoptosis driven by mitochondrial pathway is considered to be an important anti-cancer way in which Cela can induce programmed cell death in various tumor cells (Kashyap et al., 2018), such as non-small cell lung cancer cells (Fu et al., 2022), breast cancer cells (You et al., 2018), and liver cancer cells (Li et al., 2015). Mitochondrial apoptosis pathway is induced by Bcl-2 family proteins. When cells are affected by internal apoptosis stimulating factors such as reactive oxygen species (ROS), the internal mitochondrial apoptosis pathway will be activated, thus resulting in cell apoptosis (Hu et al., 2020; Zhang et al., 2021). Therefore, delivery of Cela into mitochondria is a promising strategy to further exert its apoptosis-inducing effect mediated by mitochondrial pathway and increase its anti-cancer efficacy.

Triphenylphosphine (TPP), a nontoxic molecule with delocalized positive charge, has been widely used as mitochondrial targeting groups (Zielonka et al., 2017) for its strong binding affinity to the mitochondrial membrane (Huang et al., 2022). For instance, Tan et al. constructed a mitochondrial targeting micelle with TPP modified glucolipid conjugate (TPP-CSOSA) for the delivery of Cela (Tan et al., 2018). Xu et al. used TPP to modify the surface of multifunctional nanoparticle assembled by polypyrole and mesoporous silica to achieve precise control of drug release in mitochondria of tumor cells and generate a substantial amount of heat in mitochondria under NIR irradiation (Xu et al., 2020).

Significantly, obstacles in blood circulation caused by the high positive charge of TPP before reaching the mitochondria of tumor cells should be considered when constructing a mitochondrial-targeted drug delivery system. Although PEG with negative charge is a popular biocompatible polymer due to its stealth effect on blood circulation, making it usually used to shield the positive charge of cationic drug carriers, emerging clinical evidence has shown that PEGylated particles can be recognized and cleared by anti-PEG antibodies, also known as the accelerated blood clearance (ABC) phenomenon (Tagami et al., 2009). Hyaluronic acid (HA), a glycosaminoglycan commonly presented in extracellular matrix (Garantziotis & Savani, 2019), is a natural, nontoxic, non-immunogenic, biodegradable and negatively charged polymer, which can shield the positive charge of cationic nanocarriers by electrostatic action, thus preventing the cationic nanocarriers from binding to some proteins in blood circulation (Luo et al., 2019). Moreover, HA can be specifically recognized by CD44 receptors (Cluster of differentiation-44) (Saneja et al., 2018) which are overexpressed on several malignant tumor-cell surfaces, such as MCF-7, HepG2 and etc., making it a more competitive biomaterial for targeted drug delivery.

**Figure 1.**
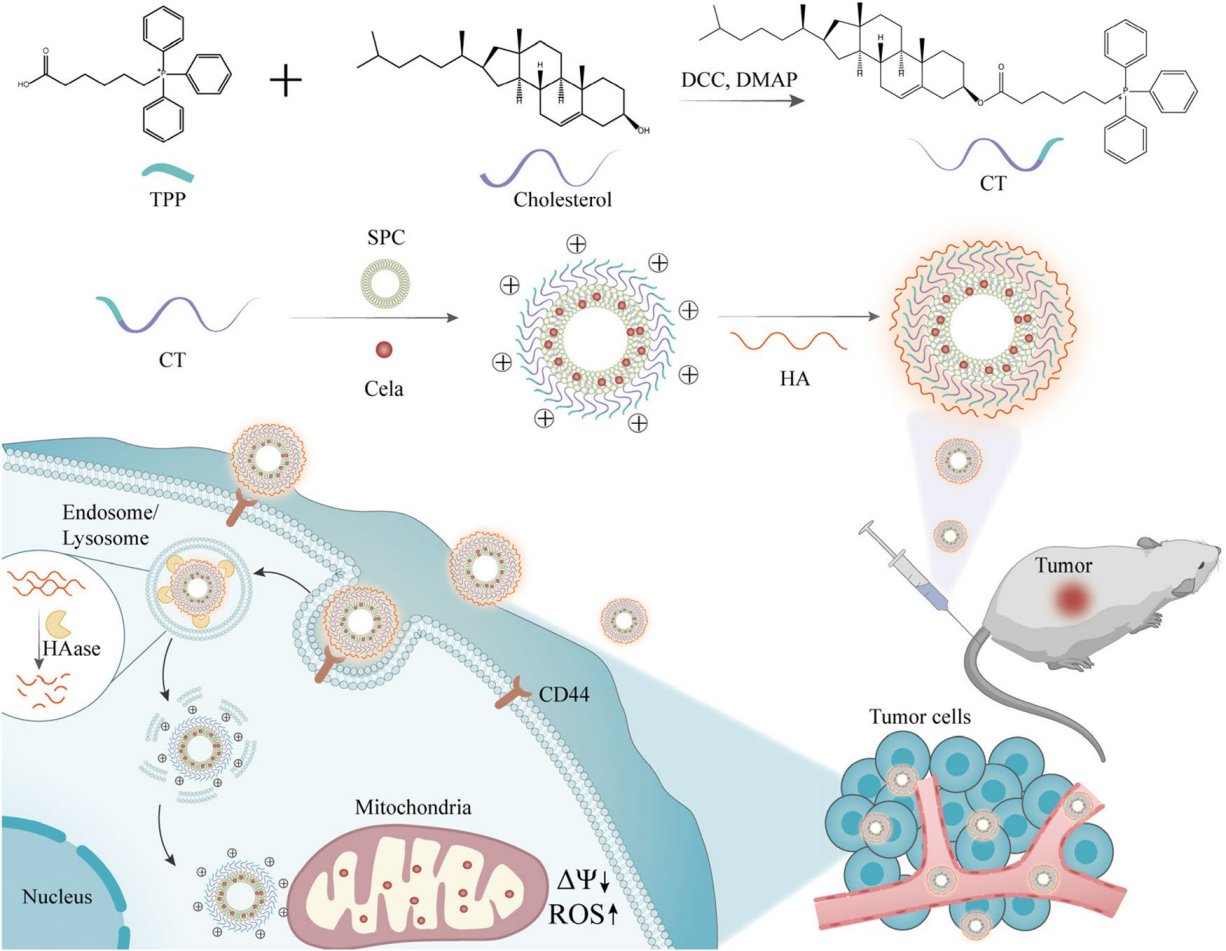
Schematic illustration of the synthesis of CT, the construction of C-TL/HA, and *in vivo* trafficking process of C-TL/HA including accumulation at tumor site, CD44 receptor-mediated endocytosis, the degradation of HA, mitochondrial targeting and apoptosis of mitochondrial pathway.

In this study, given the pivotal role of mitochondria-mediated apoptosis pathway in Cela anticancer efficacy and the obstacles in blood circulation caused by mitochondrial targeting material TPP, cholesterol linked with TPP (CT) was synthesized and assembled into mitochondria-targeted cationic liposomes with natural soybean phosphatidylcholine (SPC) for delivering Cela (Figure 1). Subsequently, negatively charged HA was electrostatically bound to the outer surface of the above Cela-loaded cationic liposomes to facilitate tumor targeting ability and stability in blood circulation. The prepared HA-coated mitochondrial targeting liposomes were expected to be stable and safe *in vivo*, would effectively aggregate around tumor cells through EPR effect (Fang et al., 2020), and would be internalized into tumor cells via the abundant CD44 receptors in the cytomembrane. Due to HA degradation by hyaluronidase in endo-lysosomes, the positive charge of TPP on liposomes would be exposed, thus targeted transporting Cela into mitochondria, triggering apoptosis of mitochondrial pathway, and ultimately killing tumor cells. To prove the effectiveness of this multistage-targeted delivery system, a series of *in vitro* and *in vivo* experiments were carried out, such as HA degradation, hemolysis rate, stability evaluation, mitochondrial localization, ROS levels and *in vivo* imaging, etc. Human liver cancer cell HepG2 with high expression of CD44 receptors and HepG2 tumor-bearing nude mice were chosen as *in vitro* and *in vivo* models to evaluate the targeting effect of this liposomes and the anticancer efficacy of Cela.

## 2. Materials and methods

### 2.1. Materials

Celastrol was supplied by Chengdu Push Biotechnology Co., Ltd. SPC was purchased from Shanghai Advanced Vehicle Technology Co., Ltd. Cholesterol and Nile Red were received from Beijing Solarbio Science & Technology Co., Ltd. (5-carboxyl pentyl) triphenyl phosphine bromide and HA were purchased from Shanghai Aladdin Biochemical Technology Co., Ltd. Coumarin 6 was obtained from Chengdu Jiaye Biological Technology Co., Ltd. The other reagents and solvents used were of analytical grade.

HepG2 and other cell lines in this study were supplied by Shanghai Institute of Biochemistry and Cell Biology, and they were cultured in high-glucose DMEM medium containing antibiotics and 10% fetal bovine serum (FBS) under conventional cell culture standard (5% CO_2_, 37 °C).

The BALB/c-nude mice were supplied by Jiangsu Jicui Yaokang Biotechnology Co. Ltd. The animal experiments were performed in compliance with Guide for the Care and Use of Laboratory Animals, approved by Hubei University of Chinese Medicine.

### 2.2. Synthesis of CT

TPP (5-carboxyl amyl triphenyl phosphorus bromide, 1 mmol) was dissolved in dichloromethane with DCC (1.2 mmol) and DMAP (1.2 mmol), and reacted for 3 h under nitrogen protection to activate carboxyl. Cholesterol (1 mmol) dissolved in dichloromethane was added drop by drop to the mixture and continuously reacted under stirring for 21 h. The end product CT was extracted by dilute hydrochloric acid, and then isolated and purified by preparative liquid chromatography. Its structure was characterized by ^1^H NMR (Advance 400, Bruker), ^13 ^C NMR (Advance 400, Bruker), FTIR (Vertex 70, Bruker), and ESI-HRMS (Q Exactive, Thermo Scientific). CDCl_3_ was used as NMR solvent. FTIR samples were prepared by dissolving CT in chloroform, dropping it onto the glass slide, and drying it under deuterium lamp. Finally, the purity of CT was analyzed by HPLC (1260 Infitiny, Agilent) performed on an InertSustain C18 (250 mm × 4.6 mm, 5 μm) column. HPLC conditions: 100% methanol; flow rate 1 mL/min; detection wavelength 287 nm.

### 2.3. Preparation and characterization of Cela-loaded mitochondrial targeting liposomes (C-TL/HA)

C-TL was prepared by the film dispersion method (Zhang et al., 2019). Briefly, Cela, SPC and CT were dissolved together in the chloroform solvent at a mass ratio of 1:30:3. After rotary evaporation, 5 mL of purified water was used to hydrate the thin lipid membrane, and then the lipid turbid solution was sonicated until it was clear and transparent. The final liposome solution (C-TL) was obtained after the unencapsulated Cela was removed with a 0.22 µm filter membrane.

C-TL/HA was obtained by adding C-TL into the HA solution at the mole ratio of 3:1 (HA:CT).

The particle size, polydispersity index (PDI) and zeta potential of C-TL and C-TL/HA were determined by Malvern particle size meter (Nano ZS90, Malvern). Morphology of liposomes was analyzed by transmission electron microscopy (JEM-1400, JEOL). The encapsulation efficiency of Cela was calculated according to the literature method (Chen et al., 2020).

### 2.4. Drug release

Dialysis was performed to survey the release profile of Cela. In brief, 1 mL of liposomes was packed in a dialysis bag (MWCO 3000) and then immersed into 40 mL of dialysis medium containing 0.1 M PBS (pH 7.4) and 1% Tween 80 (w/v). At planned time interval, 1 mL of dialysis medium was removed and replaced with 1 mL of fresh medium. The amount of Cela release was measured via HPLC as described above. The cumulative percentage of drug release (Qr) was calculated using equation (1):

(1) $$Qr(\%)=\frac{\sum_{1}^{n-1}C_{i}\times V_{1}+C_{n}\times V_{2}}{m}\times 100\%$$

Where m represents the weight of loaded Cela, V_1_ is the volume of taken buffer (1 mL), V_2_ is the total volume of dialysis fluid (40 mL), and C*_i_* denotes the Cela concentration in the *i*th sample.

### 2.5. Degradation of hyaluronic acid

The variations in zeta potential of C-TL/HA after treatment with HAase at different pH were monitored to investigate the degradation of HA. C-TL/HA solution packed in dialysis bag (MWCO 3000) was immersed into 50 mL of water bath containing different pH values with or without HAase. Zeta potential was detected by dynamic light scattering at 0, 1, 2, 3, 6 h.

### 2.6. Serum stability

Serum stability was assessed by measuring the particle size and zeta potential of C-TL and C-TL/HA after incubation with 10% FBS at different time points.

### 2.7. Hemolysis test

C-TL and C-TL/HA (1 mg/mL) were diluted to corresponding concentration respectively. The positive control group was 10 mg/mL of Triton X-100 solution representing total hemolysis, while the negative control group was normal saline without hemolysis. The OD value at 540 nm was measured with a microplate reader (Nano Drop 2000c, BIO-RAD), and the hemolysis rate (%) of liposomes at different concentrations was calculated using the following formula (2):

(2) $$\mathrm{hemolysis}\;\mathrm{rate}(\%)=\frac{{\mathrm{OD}}_{test}-{\mathrm{OD}}_{blank}}{{\mathrm{OD}}_{positive}-{\mathrm{OD}}_{blank}}$$

### 2.8. Cellular uptake

To visualize the endocytosis of liposomes in the HepG2 cells, coumarin 6 (C6) with green fluorescence was used to replace Cela without fluorescence. The preparation of C6-loaded liposomes (C6-TL and C6-TL/HA) was similar to that of Cela-loaded liposomes described above. After incubation with free C6, C6-TL, and C6-TL/HA (*n* = 4) for 4 h, the HepG2 cells were photographed by cell imaging system (EVOS FL, Thermo Fisher), and the fluorescence intensity of C6 in the cells was determined using flow cytometry (NovoCyte, ACEA).

To investigate whether the HA-coated liposomes (C-TL/HA) have targeting effect on CD44-overexpressed tumor cells, CD44-overexpressed HepG2 cells and CD44-deficient mouse liver cells AML-12 were incubated with C6-TL/HA for 4 h at 37 °C after pretreatment with free HA (1 mg/mL) for 1 h at 37 °C.

### 2.9. Cell viability

The CCK8 assay was used to evaluate the cytotoxicity of Cela-loaded liposomes (C-TL and C-TL/HA) to HepG2 cells and that of blank liposomes (TL and TL/HA) to normal human renal epithelial 293 T cells. After the cells in 96-well plates were cultured with different liposomes for 24 h, 100 µL of 10% CCK8 was added to each well, and then the absorbance values of each well were determined using a microplate reader at a wavelength of 450 nm.

### 2.10. Apoptosis

HepG2 cells in a six-well plate were incubated with free Cela and C-TL/HA, and the Cela concentration in each well was 1 μg/mL. After 24 h, the cells were washed and resuspended with PBS, and then stained by FITC and PI, respectively. The apoptosis rate of different formulations was determined using flow cytometry.

### 2.11. Localization in mitochondria

Mitochondria targeting of the liposomes in HepG2 cells was observed by confocal laser scanning microscope (CLSM, IX81-FV1000, Olympus). HepG2 cells were seeded into a CLSM dish and incubated with free C6 and C6-TL/HA for 4 h. The C6 concentration in each dish was 1 µg/mL. After cells were stained with MitoTracker Red (C1049, Beyotime Biotechnology Co., Ltd), the cell photographs were taken by CLSM.

### 2.12. ROS assay

Intracellular ROS levels were assayed using fluorescence probe DCFH-DA according to the instruction of ROS assay kit (CA1410, Beijing Solarbio Science & Technology Co., Ltd). HepG2 cells were planted in CLSM dish and incubated with free Cela and C-TL/HA. After 24 h of incubation, DCFH-DA stock solution (10 µmol/L) was added to each dish and the cells continued to be cultured for 30 min. Fluorescence photos of cells were taken by fluorescent inverted microscope (IX73, Olympus) and flow cytometry was used to detect fluorescence intensity.

### 2.13. Mitochondrial membrane potential

Mitochondrial membrane potential (ΔΨm) was detected using fluorescent probe JC-1 (M8650, Beijing Solarbio Science & Technology Co., Ltd). HepG2 cells were seeded into a CLSM dish at 37 °C for 24 h, and then incubated with free Cela and C-TL/HA for 24 h. After staining with JC-1 working solution for 20 min, the cells were washed twice with ice-cold PBS and immediately observed using fluorescent inverted microscope. Flow cytometry was used to detect fluorescence intensity of suspended cells after trypsinization.

### 2.14. Protein expression assay

The expression of mitochondrial apoptosis pathway related proteins, such as Bcl-2, Bax, Bak and Caspase-9, was detected using Western-blot method after HepG2 cells were treatment with free Cela and C-TL/HA for 24 h at a Cela concentration of 1 μg/mL. The extraction of cell total proteins and western blot immunoassay were carried out according to the literature (Chen et al., 2017). Bcl-2, Bax, Bak and Caspase-9 were probed with following antibodies: Bcl-2 Rabbit mAb (Cell Signaling Technology), Bax Mouse mAb (Wuhan Sanying Biotechnology Co., Ltd), Bak Rabbit mAb (Shenyang Wan Class Biotechnology Co., Ltd), Caspase-9 Mouse mAb (Cell Signaling Technology), respectively.

### 2.15. In vivo imaging

The right lower abdomen of each nude mouse was subcutaneously injected with 0.2 mL of cell suspension to fabricate the HepG2 tumor models, and the density in cell suspension was 2 × 10^7^ cells/mL. Vernier caliper was used to measure the length (L) and width (W) of tumors. Tumor volumes were calculated according to the formula: L × W^2^/2.

The HepG2 tumor-bearing nude mice with 100-200 mm^3^ of tumor volumes were intravenously administrated with different Nile red (NR) formulations (free NR, NR-TL and NR-TL/HA), and the injection dose of NR per mouse was 0.2 mg/kg. To further validate that the tumor targeting capability is attributed to HA coating, one group of mice were injected with NR-TL/HA after pretreatment with free HA for 1 h. The mice were anesthetized by isoflurane at the designated time points and photographed rapidly using a small animal *in vivo* imaging system (IVIS Lumina III, PerkinElmer) at the fluorescence intensity of EM530/635 nm. Additionally, at 24 h post-injection, the mice were sacrificed after anesthetized by isoflurane. Afterwards, tumors and major organs (heart, liver, spleen, lung and kidney) were harvested and photographed.

### 2.16. In vivo antitumor efficacy

HepG2 tumor-bearing nude mice were randomly separated into three groups (*n* = 5) and intravenously administrated with 5% glucose, free Cela and C-TL/HA, respectively. The Cela dose was 2 mg/kg, and the mice was administrated every other day for a total of seven times. The tumor volumes and body weights of mice were measured during administration. All mice were euthanized after treatment. Then, tumors and major organs were collected for hematoxylin and eosin staining.

### 2.17. Statistical analysis

Experimental results were presented as mean ± standard deviation (SD). The Prism 7.0 software (GraphPad Inc) was used to analyze experimental data, and comparisons were performed using Student’s t-test and one-way ANOVA. Statistical significance was considered at *p < 0.05, **p < 0.01, and ***p < 0.001.

## 3. Results and discussion

### 3.1. Synthesis and characterization of CT

**Figure 2.**
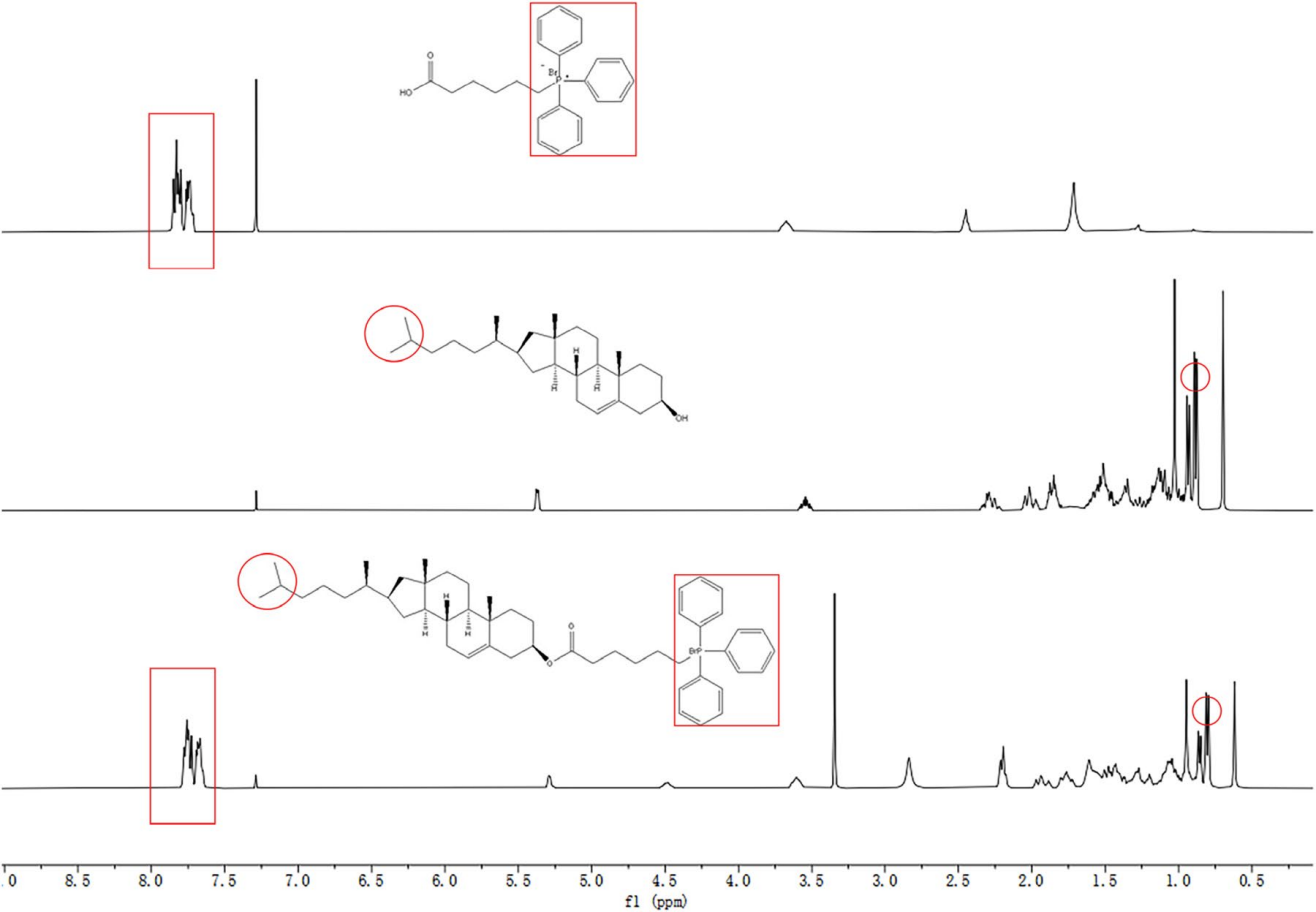
^1^H NMR spectra of TPP, Cholesterol and CT in CDCl_3_ solution. The characteristic peaks are pointed out by red circle and red rectangle.

To fabricate mitochondria-targeted liposomes, a new mitochondrial targeting material (CT) was synthesized through conjugating TPP and cholesterol, an essential membrane stabilizer of liposomes. The synthetic route of CT is shown in Figure 1. And the result of ^1^H NMR was shown in Figure 2 and S1(A). The peak at 0.82 ppm was attributed to the protons of -CH_2_, consistent with that of cholesterol, and the characteristic peaks at 7.68 ppm belonged to the protons of phenyl groups from TPP. Furthermore, ^13 ^C NMR (Figure S1(B)) and FTIR (Figure S1(C)) proved that TPP and cholesterol were linked by ester bond. The molecular ion peaks in HRMS (Figure S1(D)) suggested that the molecular mass of CT was in agreement with the theoretical value, which amply verified the successful synthesis of CT. The purity of CT was determined to be 98.36% by HPLC (Figure S1(E)). ^1^H NMR (CDCl_3_, 400 MHz) δ (ppm) 7.82 − 7.68 (m, 9H), 7.67 − 7.56 (m, 6H), 5.26 (d, *J* = 5.3 Hz, 1H), 4.53 − 4.38 (m, 1H), 3.65 − 3.50 (m, 2H), 2.17 (t, *J* = 7.5 Hz, 4H), 1.98 − 1.85 (m, 2H), 1.80 − 1.67 (m, 3H), 1.59 − 1.15 (m, 17H), 1.10 − 0.95 (m, 7H), 0.94 − 0.87 (m, 5H), 0.87 − 0.80 (m, 4H), 0.78 (d, *J* = 6.6 Hz, 6H), 0.59 (s, 3H).

### 3.2. Preparation and characterization of Cela-loaded liposomes

**Figure 3.**
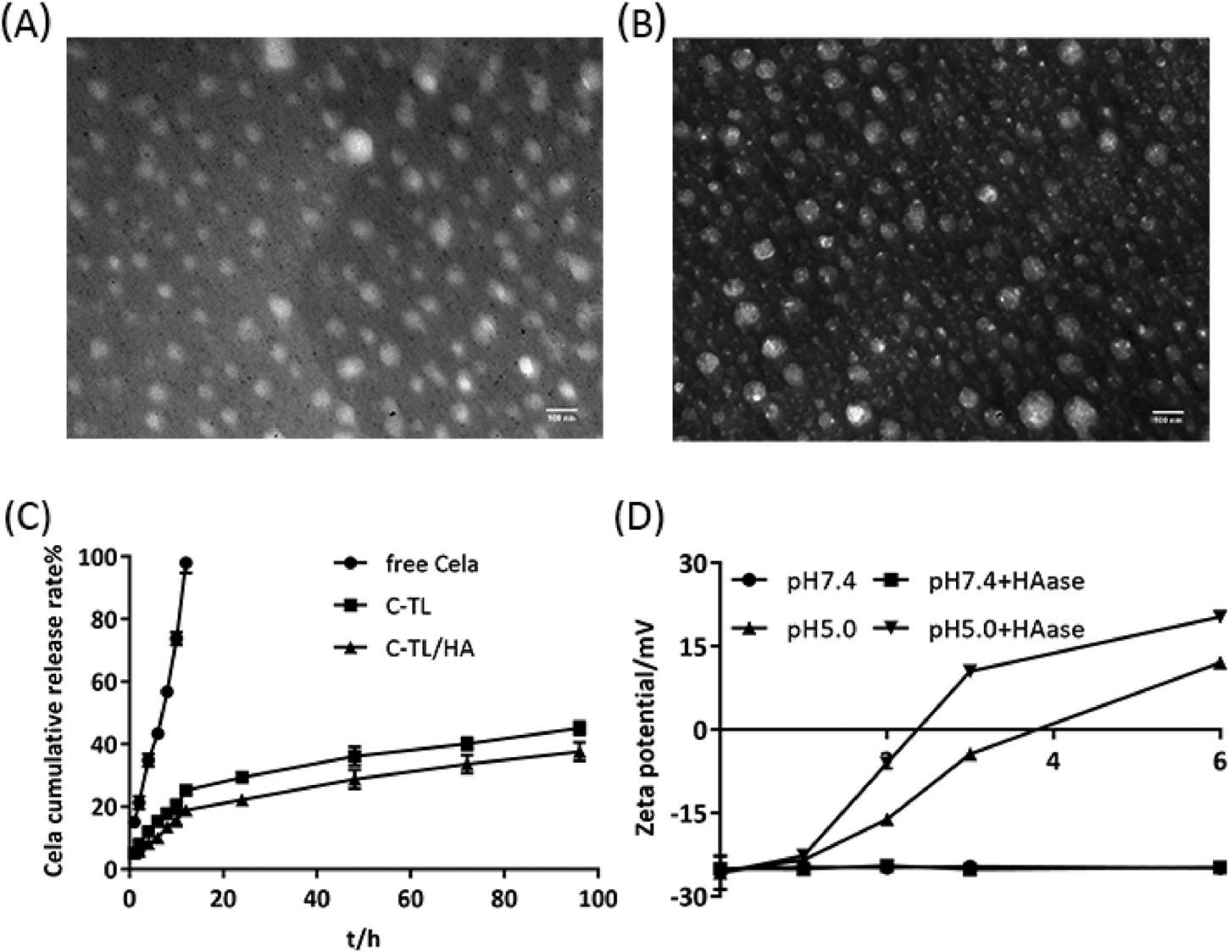
TEM images of C-TL (A) and C-TL/HA (B). (C) *In vitro* cumulative release rate of Cela. (D) Change in the zeta potential of C-TL/HA after incubation with and without HAase (1 mg/mL) at different pH (pH 7.4 and 5.0) over time.

To realize mitochondria-targeted delivery of Cela, Cela-loaded mitochondrial-targeted liposomes (C-TL) were developed based on CT and SPC. Firstly, the mass ratio of CT to SPC was screened (Table S1). At the optimal ratio of 1:10, the encapsulation efficiency of Cela was reaching 99%, the particle size was lower than 100 nm (83.77 ± 1.04 nm), and the zeta potential of C-TL was strongly positive (+28.57 ± 0.53 mV), which was conducive to target mitochondrial membrane (Table 1). Then, a certain amount of HA was mixed with C-TL to develop tumor targeting liposomes (C-TL/HA) with blood circulation stability. The usage of HA was screened and the result was shown in Table S2. Under the optimum HA dosage, C-TL/HA presented a zeta potential of −23.43 ± 2.20 mV, and this noticeable change in surface charge implied that HA had been coated on the outer layer of C-TL and successfully blocked the positive charge of TPP. Furthermore, the encapsulation efficiency of C-TL/HA was similar to that of C-TL, and the particle size of C-TL/HA was slightly higher than that of C-TL (88.97 ± 1.27 nm) but still lower than 100 nm, which contributed to gathering around tumor cells via EPR effect. The micromorphology of C-TL and C-TL/HA were witnessed by TEM as a sphere with uniform size (Figure 3A and B).

**Table 1.** Characteristics of Cela-loaded liposomes (*n* = 3).

Liposomes	Size in diameter/nm	Zeta potential/mV	Encapsulation efficiency/%	Polydispersity Index
C-TL	83.77 ± 1.04	28.57 ± 0.53	99.25 ± 0.34	0.20 ± 0.01
C-TL/HA	88.97 ± 1.27	−23.43 ± 2.20	98.37 ± 0.28	0.20 ± 0.01

### 3.3. In vitro drug release

As shown in Figure 3C, the cumulative release amount of Cela in free Cela group exceeded 50% in the first 8 h and released completely before 12 h, demonstrating a fast-release profile. Conversely, the release behaviors of Cela in C-TL and C-TL/HA groups were similar, only about 18% was released in the first 8 h, and less than 50% for 96 h. These results indicated that Cela presented a slow-release characteristic after being encapsulated by TL and TL/HA, and the coating of HA did not significantly impact the *in vitro* release behavior of Cela.

### 3.4. Degradation of HA

The endo-lysosomes are abundant in Hyaluronidase (HAase) (Chen et al., 2018), so the positive charge of TPP in liposomes may be exposed in endo-lysosomes due to the degradation of HA on the outer shell of liposomes by HAase, which makes it easier to escape from endo-lysosomes and enter the cytoplasm, thus facilitating the subsequent mitochondrial targeting. Degradation of HA was investigated by observing the variations in the zeta potential of C-TL/HA after incubation with and without HAase at pH 7.4 and pH 5.0 (in endo-lysosomes) (Figure 3D). The zeta potential of C-TL/HA did not exhibit significant variations after incubation with and without HAase for 6 h at pH 7.4, indicating that it was difficult to degrade HA shell of liposomes under neutral condition, even in the presence of HAase. By comparison, after incubation with HAase for 2 h at pH 5.0, the zeta potential of C-TL/HA varied from −26 mV to near −6 mV, while it took about 4 h to change to the positive potential in the absence of HAase, which indicated that HAase rich in endo-lysosomes could accelerate the degradation of HA in the acidic environment of endo-lysosomes, thus exposing positively charged TPP on liposomes.

### 3.5. Serum stability

**Figure 4.**
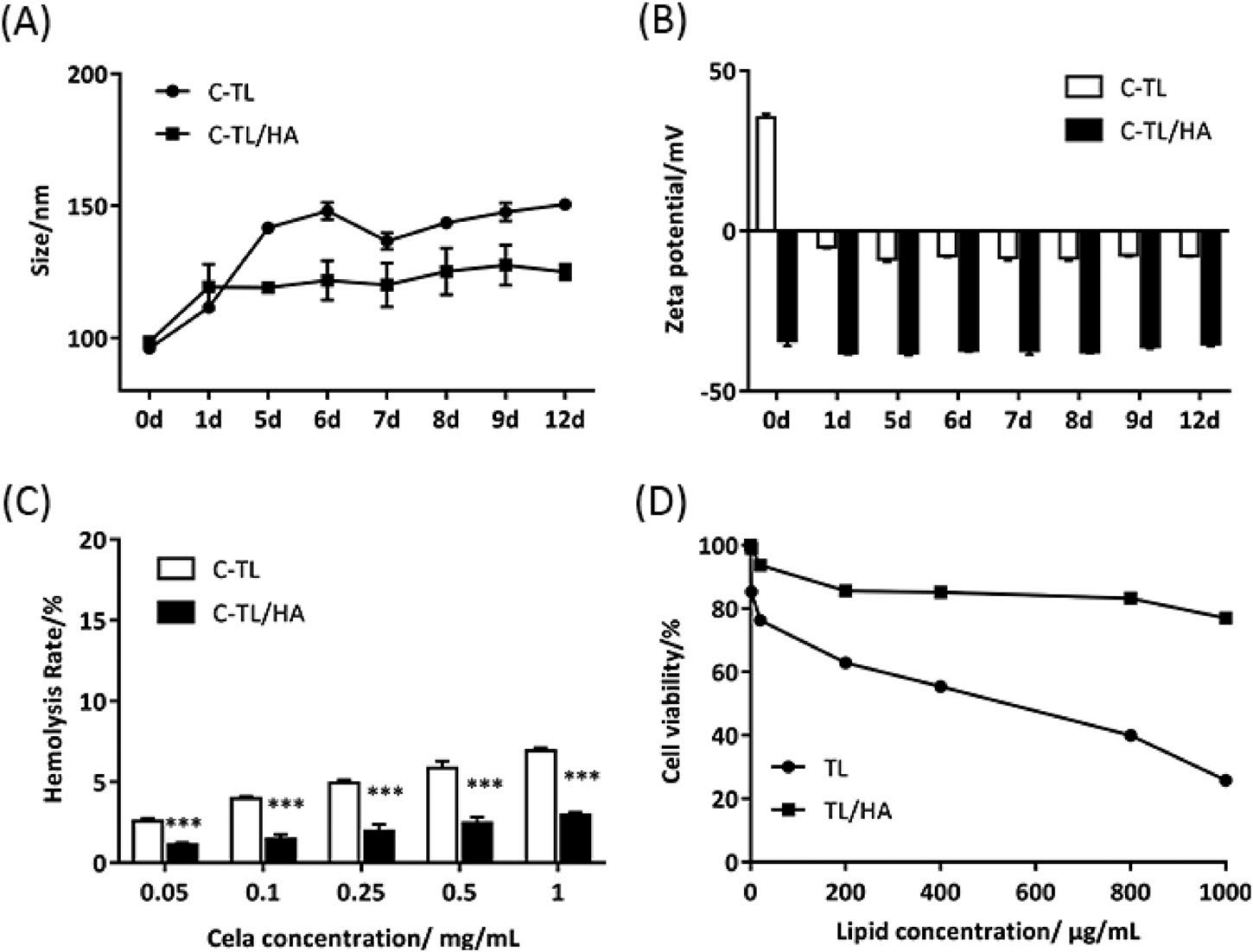
Particle size (A) and zeta potential (B) of different liposomes after incubation with 10% FBS for different time. (C) Hemolysis rate of C-TL and C-TL/HA under different Cela concentrations, ***p < 0.001, vs C-TL. (D) Cell viability of 293 T cells after treatment with TL and TL/HA for 24 h.

After incubation with 10% FBS, the particle size of C-TL increased significantly from 98.55 ± 1.65 nm to 124.97 ± 2.97 nm within 12 days, while the particle size of C-TL/HA showed a slight increase (Figure 4A). And it is worth noting that after adding 10% FBS, the zeta potential of C-TL immediately became negative, while the zeta potential of C-TL/HA did not change significantly within 12 days (Figure 4B). These results indicated that HA coating contributed to improving the stability of liposomes and avoid sharp changes of particle size and zeta potential in blood circulation. Simultaneously, the low-temperature storage experiment result also showed that C-TL/HA had relatively better low-temperature storage stability compared with C-TL (Figure S2).

### 3.6. Safety evaluation

Safety evaluation of liposomes was carried out through hemolysis test and cell viability assay of blank liposomes to normal 293 T cells. As shown in Figure 4C, when the concentration of Cela ranged from 0.05 mg/mL to 1 mg/mL, the hemolysis rate of C-TL was significantly higher than that of C-TL/HA (*p* < 0.001), illustrating that it was easier for C-TL to destroy red blood cells due to the strong positive charge.

Since the high positive surface charge of TPP may bring about some obstacles, such as inflammatory response and so on (Wang et al., 2021), which further lead to cell death, the cytotoxicity of blank liposomes (TL and TL/HA) to normal cells was investigated. As shown in Figure 4D, TL showed a higher cytotoxicity to normal 293 T cells within 24 h compared with TL/HA, indicating that the electrostatic binding of HA to TPP-modified liposomes effectively alleviated the toxicity caused by TPP. In addition, it was observed that when the lipid concentration reached 1000 µg/mL, more than 80% of the cells in TL/HA group still survived within 24 h, proving that TL/HA as a drug delivery carrier might have better *in vivo* safety.

### 3.7. Cellular uptake

**Figure 5.**
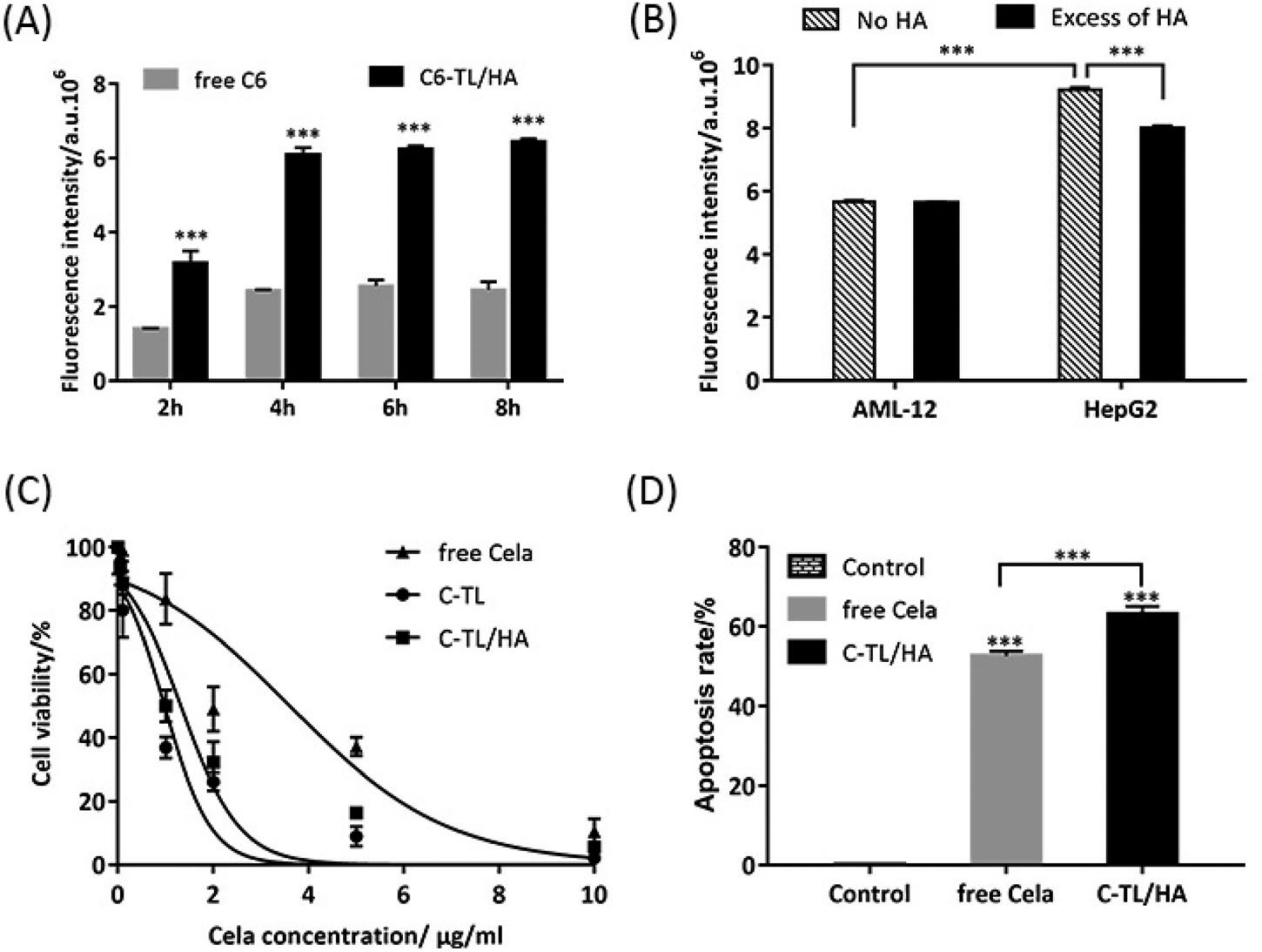
(A) Fluorescence intensity change in HepG2 cells after incubation with free C6 and C6-TL/HA over time, ***p < 0.001, vs free C6. (B) Fluorescence intensity in AML-12 and HepG2 cells incubated with C6-TL/HA for 4 h after 1 h of HA pretreatment and non-pretreatment, ***p < 0.001. (C) Cell viability after HepG2 cells were incubated with different formulations for 24 h. (D) Apoptosis rate analysis of HepG2 cells after 24 h of incubation with different formulations, ***p < 0.001, vs Control.

To quantitatively evaluate the intracellular concentration of Cela in CD44 receptor-overexpressed HepG2 cells after incubation with different formulations, liposoluble fluorescent probe C6 was loaded by TL/HA to substitute Cela. As shown by the results in Figure 5A, the fluorescence intensity of free C6 and C6-TL/HA groups both increased with increasing incubation time, but maintained relatively stable after incubation for more than 4 h. Especially, the fluorescence intensity of C6-TL/HA group was significantly higher than that of free C6 group at every incubation time point (*p <* 0.001), which implied that HA-coated mitochondria targeting liposomes (TL/HA) efficiently delivered C6 into cells and sharply increased intracellular concentration of C6.

To further demonstrate that the higher amount of cellular uptake in C6-TL/HA group may be associated with CD44 receptor-mediated recognition and endocytosis, CD44 receptor-deficient mouse liver AML-12 cells were employed to compare the cellular uptake of C6-TL/HA after pretreatment with excessive HA for 1 h and the results were shown in Figure 5B. Firstly, the fluorescence intensity of HepG2 cells was 1.6 times higher than that of AML-12 cells (*p* < 0.001) after being incubated with C6-TL/HA for 4 h, which illustrated that HA-coated liposomes facilitated the recognition and cell invasion mediated by CD44 receptor abundant on the surface of HepG2 cells. Secondly, there was no difference in fluorescence intensity between HA-pretreated and non-pretreated AML-12 cells. In sharp contrast, the fluorescence intensity of HepG2 cells was significantly different (*p* < 0.001), indicating that excessive HA pretreatment could competitively bind CD44 receptors highly expressed on the surface of HepG2 cells, thus attenuating the uptake amount of HepG2 cells to C6. Based on the above analysis, it is believable that HA-coated mitochondrial targeting liposomes as a Cela delivery system may enhance the uptake amount of HepG2 cells to Cela, and that the cellular uptake mechanism mainly relied on CD44 receptor-mediated endocytosis pathway.

### 3.8. Cell viability assay

The anti-proliferative activities of C-TL, C-TL/HA and free Cela on HepG2 cells were assessed by CCK8 method, and the results were shown in Figure 5C. C-TL/HA group displayed obviously stronger anti-proliferative effect than free Cela group, and its IC50 value was 1.32 µg/mL, about one third of that of free Cela group, which may be related to the higher cellular uptake of Cela mediated by CD44 receptor. The IC50 value of C-TL group (0.98 µg/mL) was slightly lower than that of C-TL/HA, which might be caused by the superposition of material toxicity and drug toxicity.

### 3.9. Apoptosis

As it has been confirmed that Cela can play an apoptosis inducing role in a variety of tumor cells (Medatwal et al., 2020; Xiao et al., 2022), Annexin V/PI staining was adopted to evaluate the apoptosis-inducing effect of free Cela and C-TL/HA on HepG2 cells. As shown in Figure 5D, the apoptosis rate of C-TL/HA group was significantly higher than that of free Cela group (*p* < 0.001), indicating that the enhanced proapoptotic effect of C-TL/HA might be related to the higher cellular uptake of Cela and the effective initiation of apoptosis induced by mitochondrial pathway.

### 3.10. Mitochondria targeting and mitochondrial apoptosis pathway

**Figure 6.**
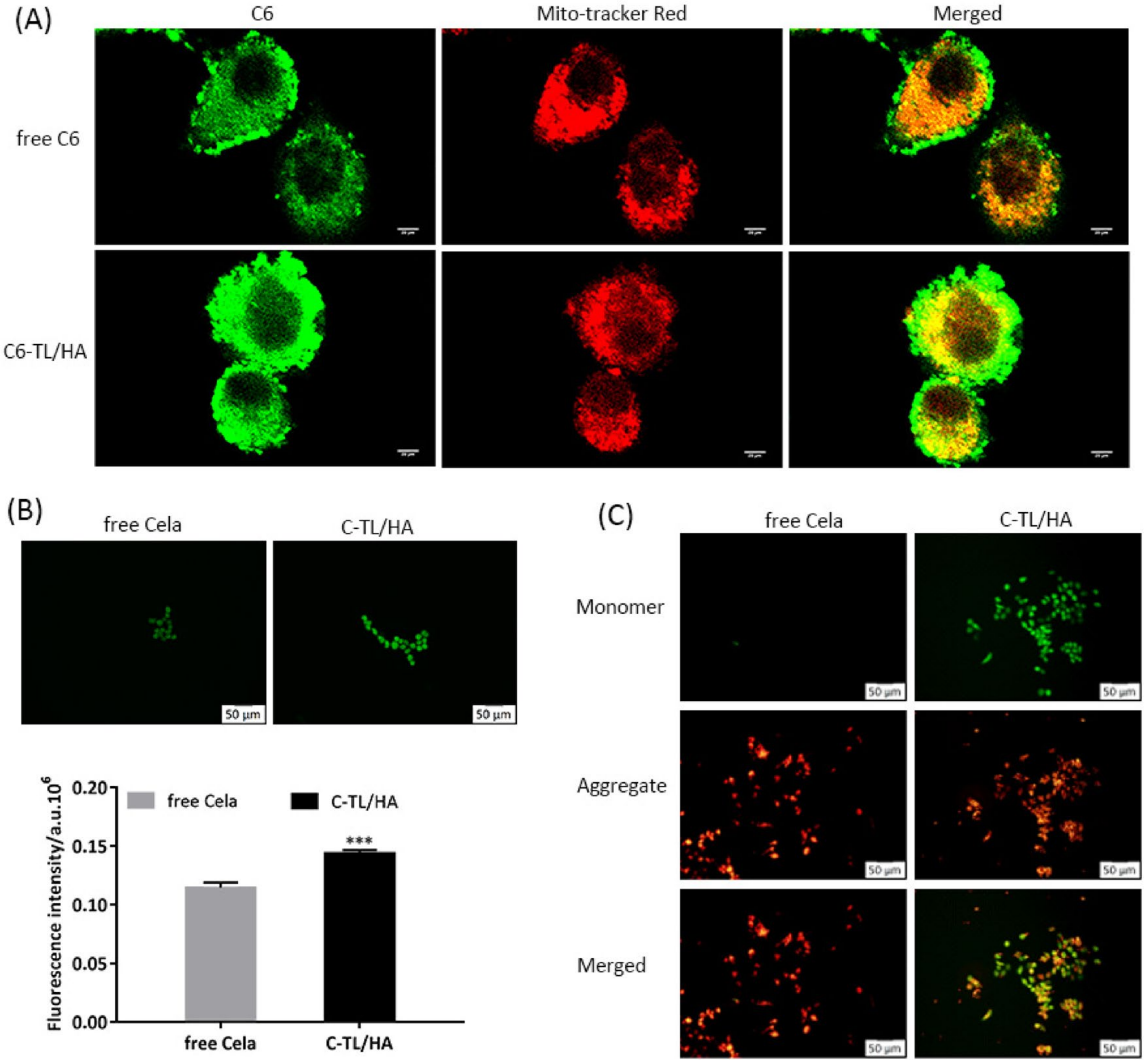
(A) CLSM images of HepG2 cells after 4 h of incubation with free C6 and C6-TL/HA. The mitochondria were stained with MitoTracker Red. (B) Comparison of the ROS levels in HepG2 cells after being treated with free Cela and C-TL/HA. Fluorescence probe DCFH-DA showed green fluorescence and its fluorescence intensity was determined by flow cytometry, ***p < 0.001, vs free Cela. (C) Mitochondrial membrane potential analysis based on JC-1 staining after HepG2 cells were treated with free Cela and C-TL/HA.

A fluorescence double-labeling method was used to investigate the mitochondrial-targeting ability of C6-TL/HA. Apart from the green fluorescence of C6 itself, the mitochondria of HepG2 cells exhibited red fluorescence after being labeled with MitoTracker red stain. The cell images obtained by CLSM were shown in Figure 6A. After incubation with HepG2 cells for 4 h, C6-TL/HA group showed stronger green fluorescence signal than free C6 group, demonstrating effective delivery of C6 into the cytoplasm by TL/HA. Compared to the extremely weak yellow fluorescence in free C6 group, C6-TL/HA group presented the remarkable yellow fluorescence signals representing the superposition of C6 and mitochondrial signals, which implied that TL/HA successfully delivered C6 into mitochondria possibly based on the excellent affinity of TPP to the mitochondrial inner membrane. According to the above analysis, it is reasonable to infer that the exposed TPP may effectively boost the drug accumulation in mitochondria after the HA outer shell of TL/HA was degraded by enzymes.

The internal mitochondrial pathway of apoptosis may be related to high levels of ROS (Su et al., 2019), so the ROS levels in HepG2 cells were detected after incubation with different formulations. As shown in Figure 6B, after treatment with C-TL/HA, the ROS level in HepG2 cells was significantly higher than that after treatment with free Cela (*p* < 0.001), indicating that C-TL/HA could stimulate more ROS generation. Considering that ROS accumulation could further lead to the decline of ΔΨm, an important proof of mitochondrial depolarization (Tan et al., 2018), so fluorescence probe JC-1 was used to detect the change of ΔΨm. As shown in Figure 6C, the red fluorescence intensity in HepG2 cells decreased dramatically after treatment with C-TL/HA, implying that the mitochondrial membrane potentials were depolarized induced by C-TL/HA. Since mitochondrial polarization is one of the early events of apoptosis, this result also indicated the occurrence of apoptosis.

**Figure 7.**
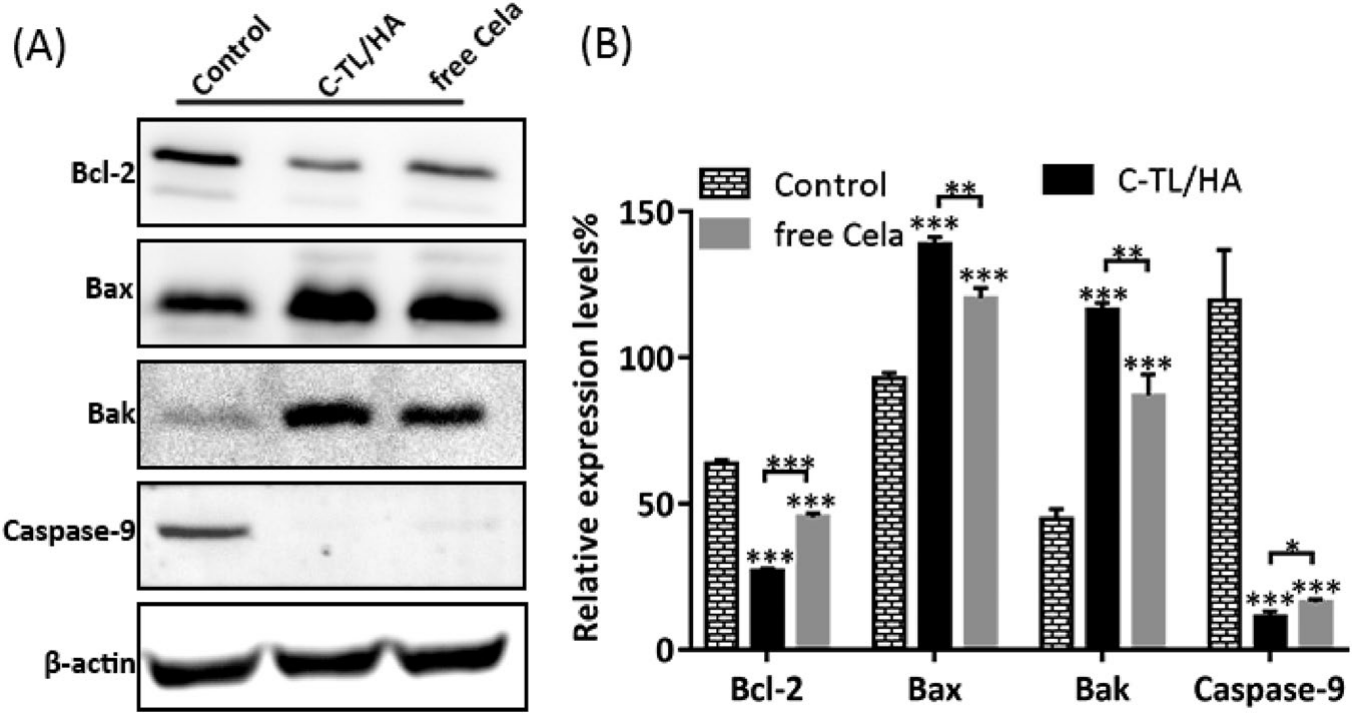
(A) Protein expression of HepG2 cells after treatment with free Cela and C-TL/HA for 24 h. (B) Relative expression levels of proteins assessed by western blot analysis, *p < 0.05, **p < 0.01, ***p < 0.001, vs Control.

It is widely known that mitochondrial apoptotic pathway is controlled and regulated by Bcl-2 protein family (Delbridge et al., 2016). As the members of Bcl-2 protein family, Bax and Bak are activated under the stimulation of apoptosis, and then accumulate on the mitochondrial outer membrane, thereby promoting the release of cytochrome c and subsequently triggering the Caspase cascade. Figure 7 depicted the expression of mitochondrial apoptotic proteins after treatment with different formulations. C-TL/HA group caused a more significant upregulation of Bak and Bax protein expression and downregulation of Bcl-2 expression when compared with the other groups, indicating that C-TL/HA held a better ability to activate mitochondrial apoptosis pathway. Additionally, the above result was also supported by the notable inhibition of Caspase-9 protein, a key initiating factor in the mitochondrial apoptosis pathway. Given that, mitochondria-specific delivery of Cela through TL/HA effectively elevated ROS generation, stimulated ΔΨm decreasing and activated mitochondrial pathway of apoptosis so as to strengthen its antitumor efficacy.

### 3.11. In vivo imaging

**Figure 8.**
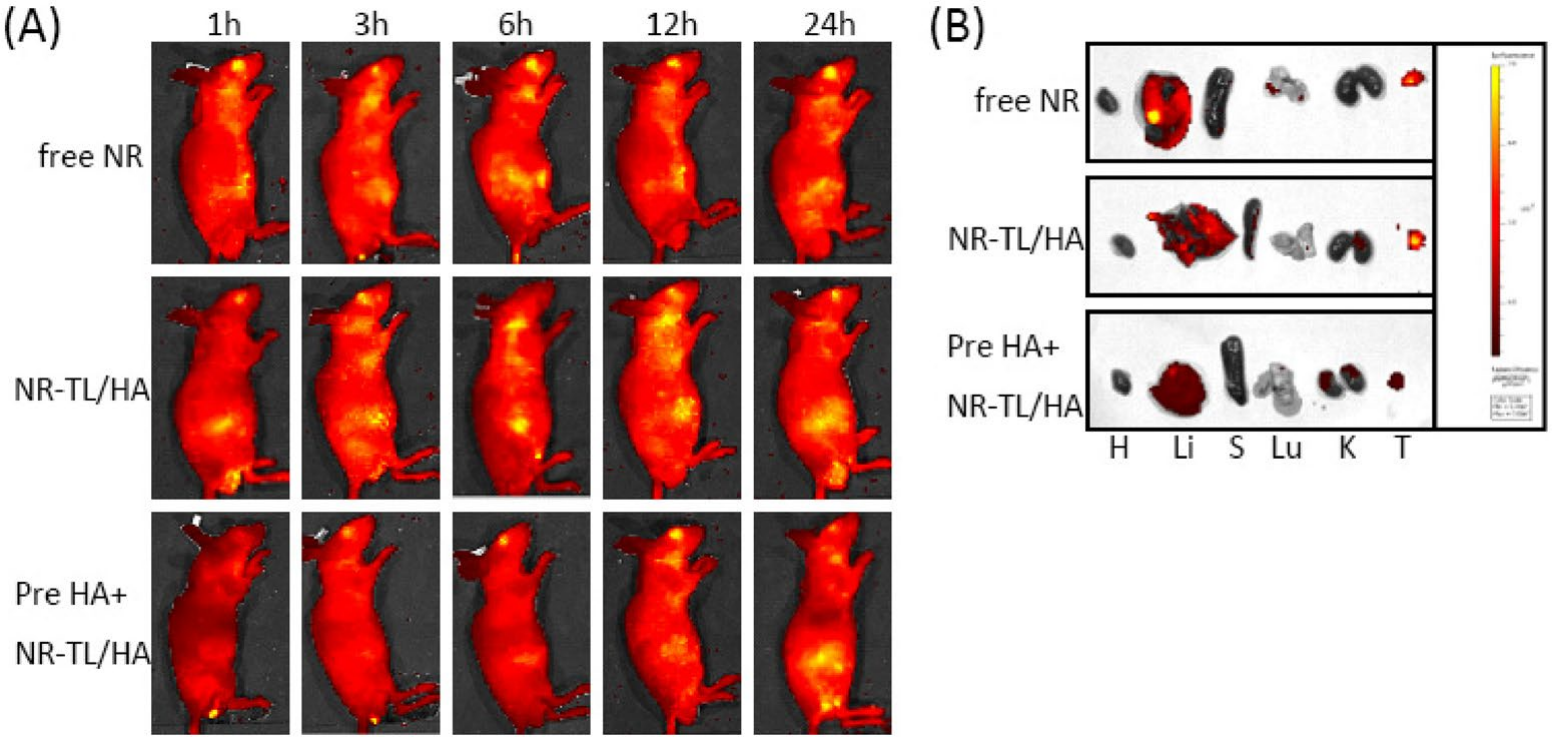
(A) *In vivo* fluorescence images of the HepG2 tumor-bearing nude mice after intravenous injection with free NR, NR-TL/HA and NR-TL/HA with pre-injection of the free HA (1 mg/kg) for 1 h. (B) *Ex vivo* fluorescence images of different tissues including heart (H), liver (Li), spleen (S), lung (Lu), kidney (K), and tumor (T) harvested at 24 h post-injection.

Near-infrared dye Nile Red was embedded in targeting liposomes TL/HA to assess its tumor targeting ability. As expected, the NR signal of NR-TL/HA was primarily concentrated in the tumor at just 1 h post-injection, and was still strong 24 h later (Figure 8A), consistent with the result of *ex vivo* imaging (Figure 8B). In contrast, free NR showed a relatively week NR signal in the tumor site during the whole observation period. In order to further confirm the HA-dependent tumor targeting effect of NR-TL/HA, free HA was pre-injected into the tumor-bearing mice to competitively block the CD44 receptors on tumors before injection of NR-TL/HA. Surprisingly, the NR signal in the tumor site could not be clearly observed until 24 h after injection, fully explaining that the HA pretreatment delayed the localization of NR-TL/HA in tumor site due to the competitive combination of pre-injected HA with CD44 receptors on the tumor surface.

### 3.12. In vivo pharmacodynamics study

The HepG2 xenograft model in BALB/c nude mice was established to evaluate the potential of C-TL/HA in targeted cancer therapy. Considering the *in vivo* safety during administration, one group of mice was treated with isotonic solution of 5% glucose (GLU) as model, and other formulations such as free Cela and C-TL/HA were prepared with 5% glucose.

**Figure 9.**
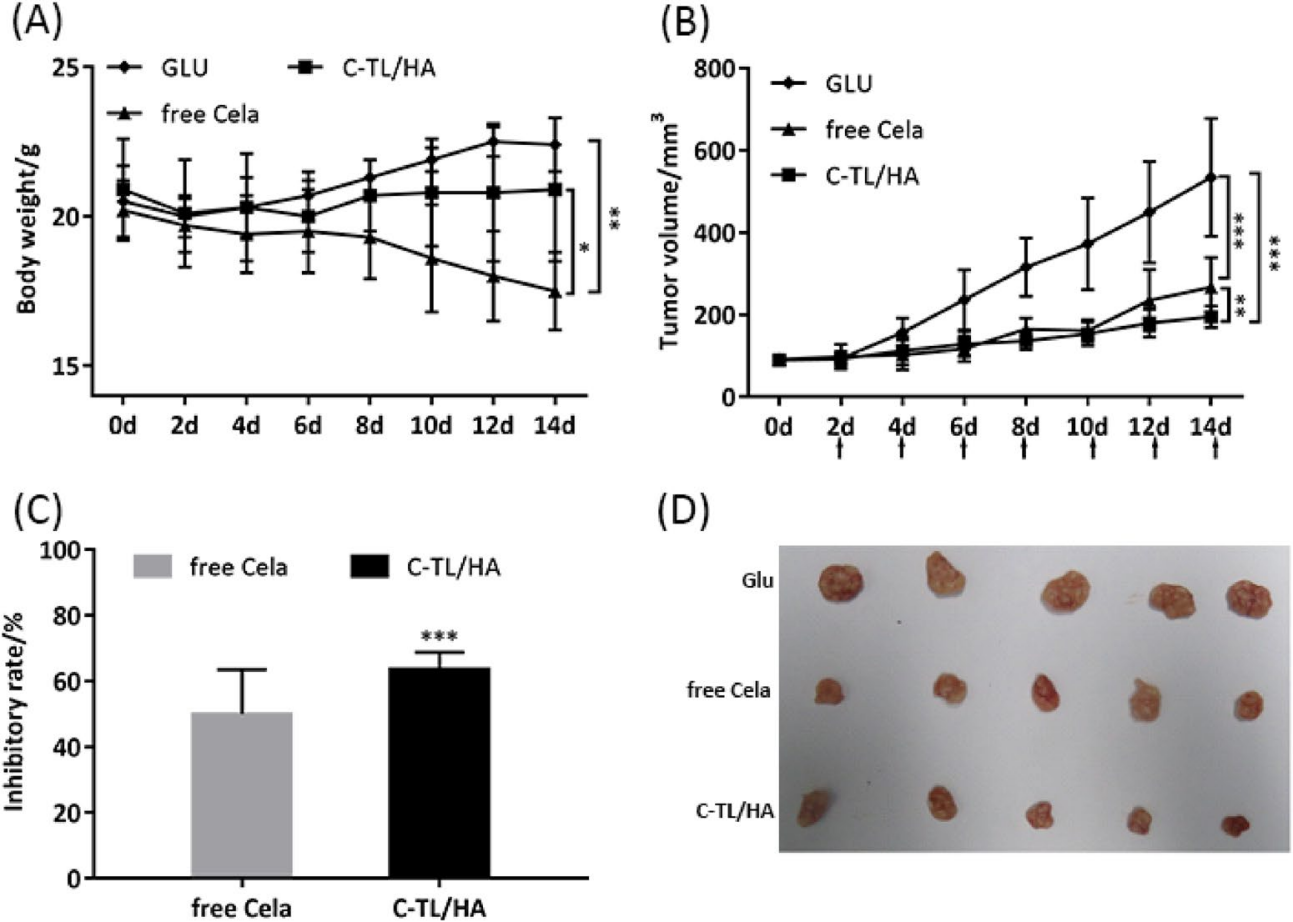
(A) Body weight variations of HepG2 tumor-bearing nude mice after treatment with free Cela, C-TL/HA and GLU, *p < 0.05, **p < 0.01. (B) Tumor growth curve of HepG2 tumor-bearing nude mice under different treatments. The arrows symbolize the time of intravenously administration. *p < 0.05, **p < 0.01, ***p < 0.001. (C) Tumor inhibitory rate analysis after treatment with free Cela and C-TL/HA, ***p < 0.001, vs free Cela. (D) Macroscopic appearance of tumors collected from HepG2 tumor-bearing nude mice after 14 days of treatment.

**Figure 10.**
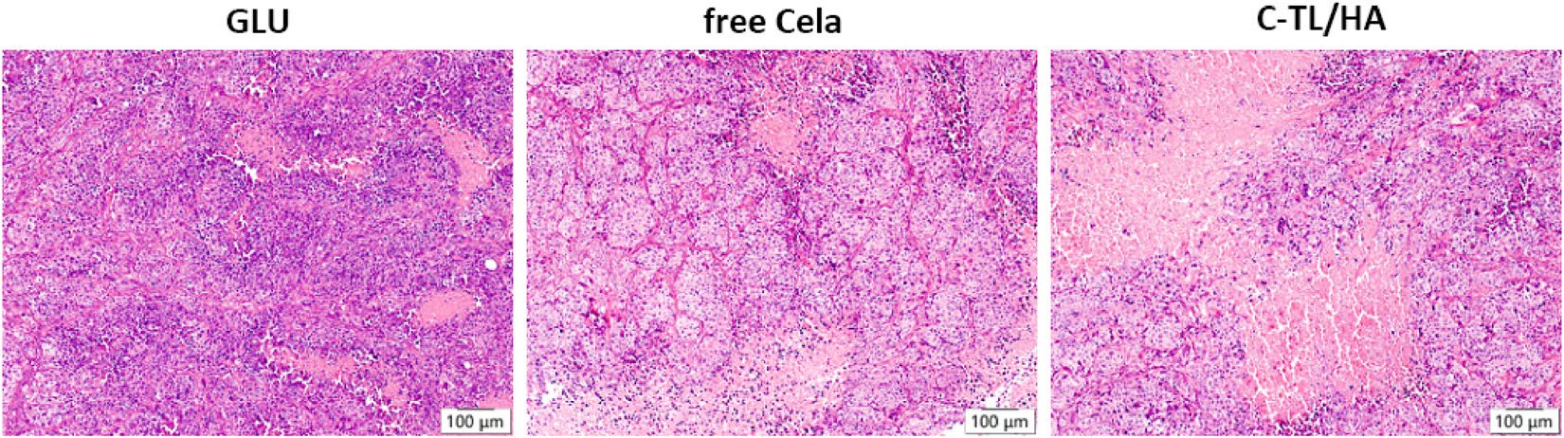
Representative images of the HE-stained tumor sections after treatment with GLU, Free Cela and C-TL/HA.

Statistically different from that of GLU (*p* < 0.01) and C-TL/HA group (*p* < 0.05), the body weights of mice treated with free Cela declined evidently, which may be related to the off-set toxicity of Cela (Figure 9A). Conversely, C-TL/HA treatment did not incur apparent weight loss, nor did it cause obvious harm to normal organs, different from clear myocardial fiber injury in free Cela treatment group (Figure S3). These results suggested that C-TL/HA had superior *in vivo* safety compared with free Cela, which might be because the HA-mediated tumor targeting effect attenuated the systemic distribution of free Cela. Figure 9B displayed the tumor volume changes in tumor-bearing mice during intravenous administration. Compared with GLU group, both Cela and C-TL/HA hampered tumor growth significantly (*p* < 0.001), but C-TL/HA displayed more evident potency to inhibit tumor growth than free Cela (*p* < 0.01), which was also evidenced by 1.4-fold higher tumor inhibition rate (Figure 9C, *p* < 0.001) and smaller stripped tumor tissues (Figure 9D). Meanwhile, it can be observed from the histological analysis results of the stripped tumor tissues that there were many aggregated blue nuclei and irregular cell shapes in GLU group, which are typical pathological features of tumor, whereas tumor necrosis occurred in free Cela and C-TL/HA groups (Figure 10). In particular, C-TL/HA group led to a significant reduction of tumor cells compared with free Cela group, suggesting that C-TL/HA had superior therapeutic efficacy against liver cancer, which could result from the increased drug uptake, precise mitochondrial-targeting and tumor-targeting effects.

## 4. Conclusions

In summary, to enhance the anti-tumor efficiency of Cela through mitochondria-mediated apoptosis and avoid the adverse effects of Cela, a new compound CT was synthesized by linking mitochondrial targeting molecule TPP and cholesterol. Then, novel liposomes were fabricated using CT and SPC for the mitochondria-targeted delivery of Cela. Negatively charged polysaccharide HA was coated on the outer layer of these liposomes to neutralize the strong positive charge caused by TPP and obtain HA-mediated tumor targeting ability. *In vitro* experimental data analysis showed that HA-coated mitochondrial-targeting liposomes not only gained high encapsulation efficiency, advanced stability and safety in blood circulation and enhanced cellular uptake of Cela, but also successfully achieved mitochondria localization, thereby inducing mitochondria-mediated apoptosis and tumor cell death. More importantly, *in vivo* analysis found that this multistage-targeted delivery system realized effective tumor-targeted delivery, elicited favorable tumor inhibition response, and in particular mitigated the systemic toxicity of free Cela, thus proving to be a promising method that can be used to overcome challenges in clinical applications of Cela.

## Supplementary Material

Supplemental Material

## Supporting materials

Characterization of CT; Screening the mass ratios of CT:SPC; Screening the mole ratios of HA:CT; Storage stability of C-TL and C-TL/HA at 4 °C; Representative images of the HE-stained normal tissues after treatment.
