# Supplementary material for: The development and evaluation of hyaluronic acid coated mitochondrial targeting liposomes for celastrol delivery
Source: Drug Deliv. 2023 Jan 4;30(1):2162156. doi: 10.1080/10717544.2022.2162156 (PMC9828745; doi:10.1080/10717544.2022.2162156)
Supplement: Supplemental Material [file IDRD_A_2162156_SM8843.doc]

**Supplemental materials**

**The development and evaluation of hyaluronic acid coated mitochondrial targeting liposomes for celastrol delivery**

Simeng Xiao,^a^ Siying Huang,^a^ Xiaojing Yang,^a^ Yujie Lei,^c^ Mingxiang Chang,^d^ Junjie Hu,^a^ Yan Meng,^a^ Guohua Zheng,^b,*^ Xinyan Chen^a,*^

^a^Pharmacy Faculty, Hubei University of Chinese Medicine, Wuhan 430065, China

^b^Key Laboratory of Chinese Medicine Resource and Compound Prescription, Ministry of Education, Hubei University of Chinese Medicine, Wuhan 430065, China

^c^Pharmacy Department, Wuxue No.1 People’s Hospital, Wuxue 435400, China

^d^Laboratory of Cell and Molecular Biology, Hubei Hospital of Traditional Chinese Medicine, Wuhan 430060, China

Corresponding Authors:

*Xinyan Chen

Phone: +86 18186220641

E-mail: chenxy_328@163.com

Address: Pharmacy Faculty, Hubei University of Chinese Medicine, Wuhan 430065, China

*Guohua Zheng

Phone: +86 13627108243

E-mail: zgh1227@sina.com

Address: Key Laboratory of Chinese Medicine Resource and Compound Prescription, Ministry of Education, Hubei University of Chinese Medicine, Wuhan 430065, China

1 Characterization of CT


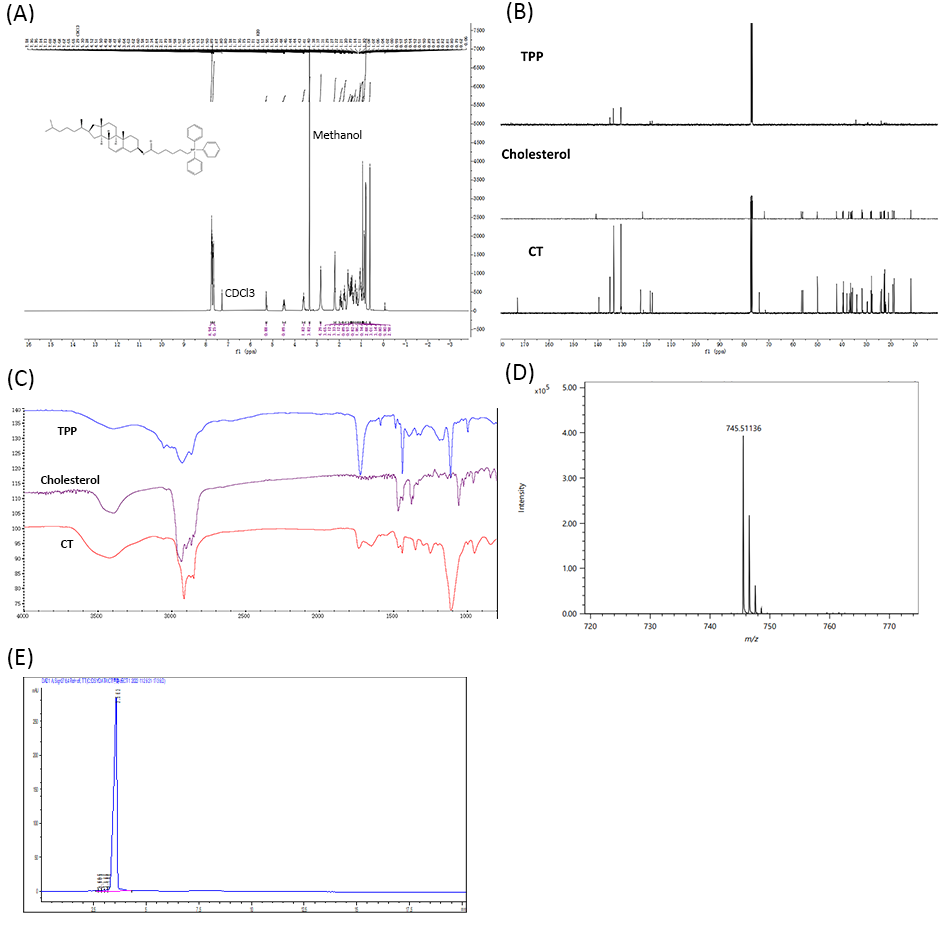
**Figure S1.** (A) ^1^H NMR spectrum of CT in CDCl_3_ solution. (B) ^13^C NMR spectra of TPP, Cholesterol and CT in CDCl_3_ solution. (C) FTIR spectra of TPP, Cholesterol and CT. (D) HRMS spectrum of CT. (E) HPLC chromatogram of CT. Column: InertSustain C_18_ (250 mm×4.6 mm, 5 μm). Mobile phase: 100% methanol. Flow rate: 1 mL/min. Detection wavelength: 287 nm.

The compound CT was characterized by ^1^H NMR, ^13^C NMR, fourier transform infrared spectroscopy (FTIR) and high-resolution mass spectrometry (HRMS). Moreover, high performance liquid chromatography (HPLC) was used to detect the purity of CT. As shown in Figure S1(A), the chemical shifts of 0.82 ppm and 7.68 ppm separately denoted the protons of -CH_2_ in cholesterol and the protons of phenyl groups in TPP, which proved the successful synthesis of CT. ^13^C NMR results (Figure S1(B)) clearly showed that the [carbonyl](javascript:;) signal in TPP shifted from 176 ppm to 173 ppm in CT, which indicated that cholesterol was linked to TPP by ester bond. And compared with the stretching vibration band of carboxyl group of TPP at 1692 cm^-1^, the stretching vibration band of carbonyl group of CT obviously increased to 1737 cm^-1^ (Figure S1(C)), which also demonstrated the successful formation of ester bond. ESI-HRMS was applied to detect the mass-to-charge ratio of CT (Figure S1(D)). With the loss of bromine ion under the attack of positive ion mode, the m/z value changed to 745.51 (C_51_H_70_O_2_P^+^), indicating that the molecular mass of CT analyzed by HRMS was consistent with its theoretical value (824.43). The purity of CT was estimated to be 98.36% by HPLC (Figure S1(E)).

2 Screening the mass ratios of CT:SPC

**Table S1.**

Physicochemical properties of C-TL at the different mass ratios of CT:SPC (n=3).

| CT:SPC | Size in diameter/nm | Zeta potential/mV | Encapsulation efficiency/% | Polydispersity Index |
| --- | --- | --- | --- | --- |
| 1:12 | 124.80±4.12 | 34.80±1.06 | 92.05±0.53 | 0.28±0.01 |
| 1:10 | 115.37±3.65 | 27.87±0.25 | 99.51±0.28 | 0.30±0.01 |
| 1:8 | 103.80±1.65 | 28.30±0.20 | 97.59±0.47 | 0.23±0.01 |
| 1:6 | 122.60±0.80 | 43.57±0.47 | 93.65±0.20 | 0.30±0.01 |

When the mass ratio of CT to SPC was 1:10, the encapsulation efficiency of C-TL was higher than that of other mass ratios (Table S1). Additionally, the moderate positive potential of C-TL at this mass ratio had sufficient mitochondrial targeting capacity, and did not require excessive HA to shield. Therefore, the optimum mass ratio of CT to SPC was 1:10.

3 Screening the mole ratios of HA:CT

**Table S2.**

**Physicochemical properties of C-TL/HA at the different** **mole ratios of HA:CT (n=3).**

| HA:CT | Size in diameter/nm | Zeta potential/mV | Polydispersity Index |
| --- | --- | --- | --- |
| 1:1 | 61.81±0.20 | 2.88±0.36 | 0.34±0.00 |
| 2:1 | 73.84±2.64 | -1.05±0.60 | 0.28±0.02 |
| 3:1 | 88.97±1.27 | -23.43±2.20 | 0.30±0.01 |
| 4:1 | 108.95±0.85 | -23.63±0.72 | 0.38±0.02 |

Obstacles in blood circulation caused by the high positive charge of TPP before reaching the mitochondria of tumor cells were settled by electrically binding negatively charged HA. As shown in Table S2, when the mole ratio of HA:CT was 1:1, the zeta potential of C-TL/HA was still positive. When the mole ratio was 3:1, the zeta potential of C-TL/HA was -23.43±2.20 mV, a suitable potential for liposomes to maintain stable in solution. However, when the mole ratio of HA:CT was 4:1, the particle size of C-TL/HA increased obviously from about 90 nm to about 110 nm. Hence, the optimum mole ratio of HA: CT was 3:1.


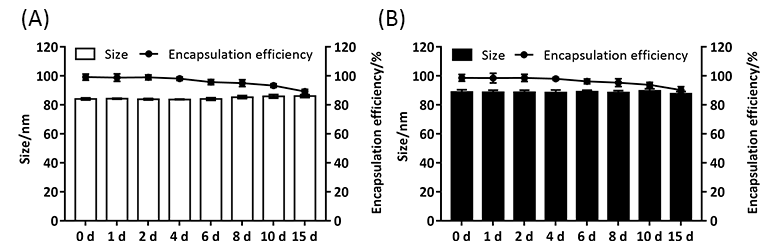
4 Storage stability of C-TL and C-TL/HA at 4 °C

**Figure S2.** Partical size and encapsulation efficiency of C-TL (A) and C-TL/HA (B) after storage at 4 ℃ for different days.

Within 15 days, the particle sizes of C-TL and C-TL/HA did not change significantly, but the encapsulation efficiency both decreased slightly. However, the encapsulation efficiency of C-TL/HA was still above 90% after 15 days of storage, while C-TL dropped below 90%, indicating that C-TL/HA had a relatively better low-temperature storage stability.

5 Representative images of the HE-stained normal tissues after treatment


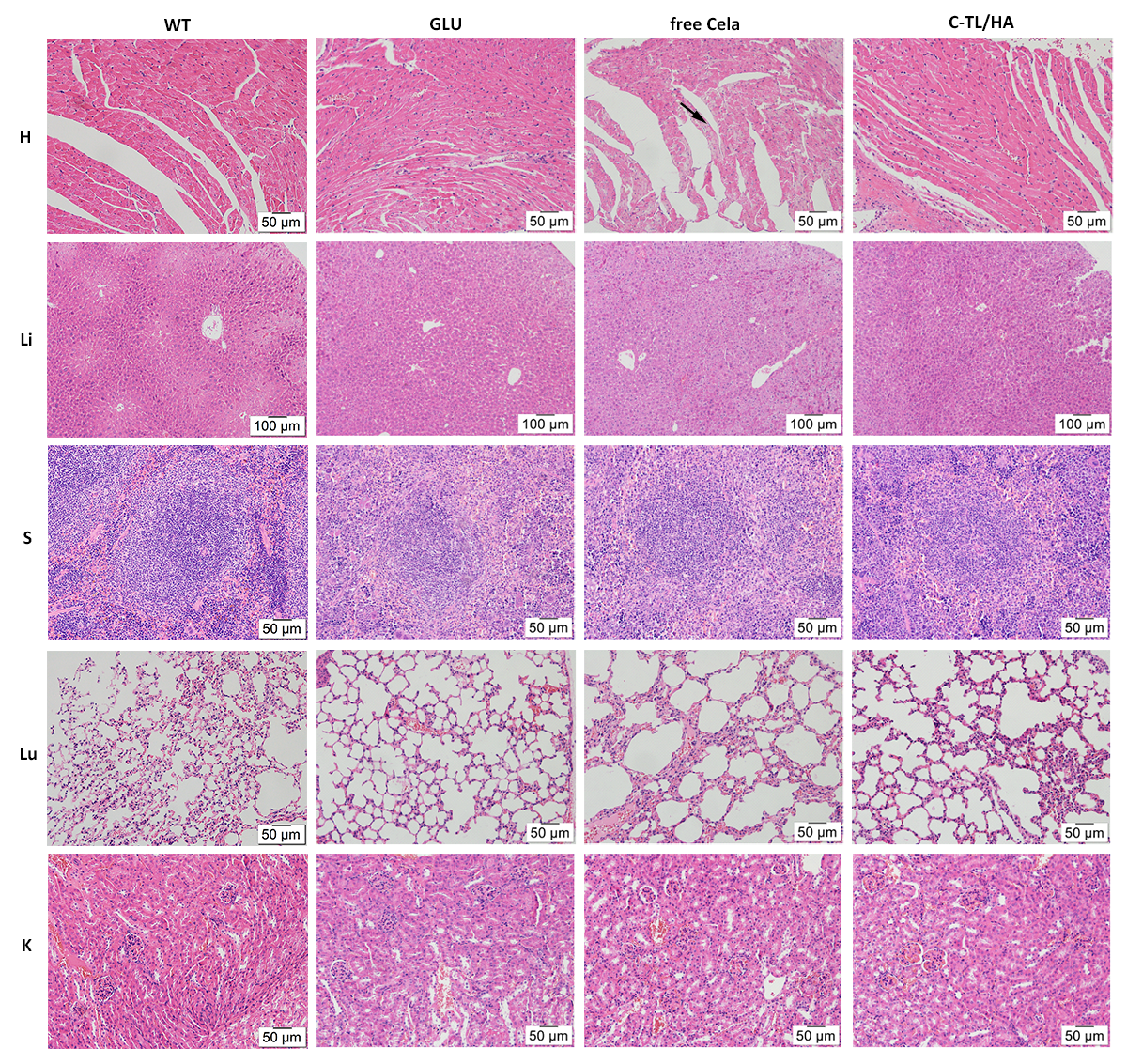
**Figure S3.** Representative images of the HE-stained heart (H), liver (Li), spleen (S), lung (Lu) and kidney (K) sections after HepG2 tumor-bearing nude mice were treated with GLU, free Cela and C-TL/HA. The black arrow denotes the damaged myocardial fibroblasts.

The pathological sections of major organs were observed to further analyse whether C-TL/HA could effectively attenuate the toxicity of Cela to normal viscera. Different from the apparent myocardial fiber injury led by 14 days of free Cela treatment, C-TL/HA did not cause obvious damage to heart, liver and other normal organs, proving that C-TL/HA treatment could mitigate the systemic toxicity of free Cela.
